# Supplementary material for: Sustained growth of sulfur hexafluoride emissions in China inferred from atmospheric observations
Source: Nat Commun. 2024 Mar 5;15:1997. doi: 10.1038/s41467-024-46084-3 (PMC10915133; doi:10.1038/s41467-024-46084-3)
Supplement: Supplementary file 1 — Supplementary Information [file 41467_2024_46084_MOESM1_ESM.pdf]

Supplementary Information for: Sustained growth of sulfur hexafluoride emissions in China inferred from atmospheric observations

Minde An[1,2,3*], Ronald G. Prinn[1], Luke M. Western[3,4], Xingchen Zhao[2], Bo Yao[5,6*], Jianxin Hu[2], Anita L. Ganesan[1,7], Jens Mühle[8], Ray F. Weiss[8], Paul B. Krummel[9], Simon O'Doherty[3], Dickon Young[3], Matthew Rigby[1,3]

1 Center for Global Change Science, Massachusetts Institute of Technology, Cambridge, MA, 02139, USA

2 College of Environmental Sciences and Engineering, Peking University, Beijing, 100871, China

3 School of Chemistry, University of Bristol, Bristol, BS8 1TS, UK

4 Global Monitoring Laboratory, National Oceanic and Atmospheric Administration, Boulder, CO, 80305, USA

5 Department of Atmospheric and Oceanic Sciences & Institute of Atmospheric Sciences, Fudan University, Shanghai, 200438, China

6 Meteorological Observation Centre of China Meteorological Administration (MOC/CMA), Beijing, 100081, China

7 School of Geographical Sciences, University of Bristol, Bristol, BS8 1SS, UK

8 Scripps Institution of Oceanography, University of California San Diego, La Jolla, CA, 92093, USA

9 Climate, Atmosphere and Oceans Interactions, CSIRO Environment, Aspendale, Victoria 3195, Australia

* **Corresponding authors**:

Bo Yao: yaobo@fudan.edu.cn；Minde An: mindean@mit.edu

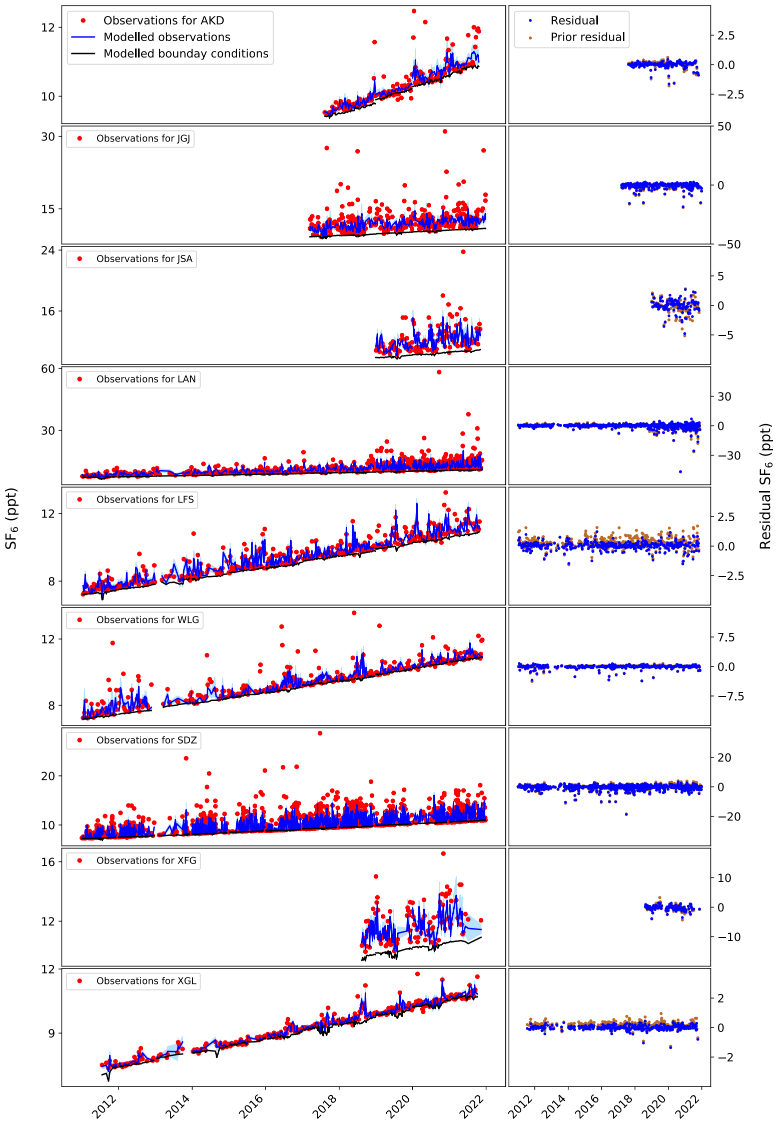

Fig. 1 Observations and simulations of SF_6 mole fractions in China. The left panels present the observed SF_6 mole fractions (red dots, after re-sampling and filtering) from each site, compared to the corresponding simulated mole fractions (blue line) and their uncertainties (blue shading) using the a posteriori emissions. The black lines in the left panels are the mean posterior predictive modelled baseline mole fractions after the inversion. The right panels present the residuals of modelled minus observed SF_6 mole fractions using the derived a posteriori emissions (blue dots) and a priori emissions (brown dots). We observed substantial enhancements of SF_6 atmospheric mole fractions above baseline levels at the measurement sites across different regions of China,

including AKD in the northwest, LAN in the east, SDZ in the north, JGJ in the southwest and others, suggesting that significant SF_6 emissions exist throughout China.

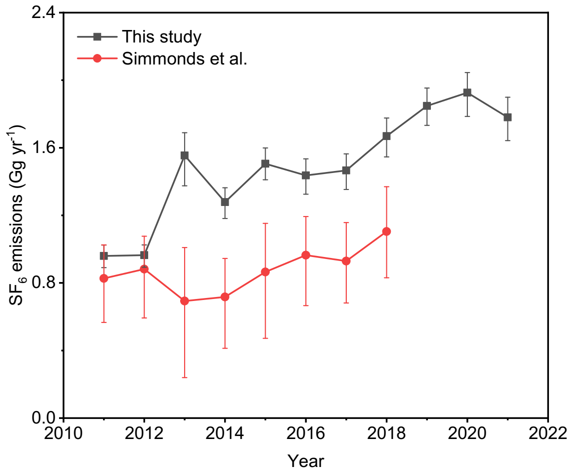

Fig. 2 SF_6 emissions in eastern China derived in this study compared to Simmonds et al.[1]. Eastern China includes the provinces of Liaoning, Beijing, Hebei, Tianjin, Shandong, Jiangsu, Anhui, Shanghai and Zhejiang, as defined by Simmonds et al.[1]. The error bars represent the 68% uncertainty intervals (or 1-sigma uncertainties).

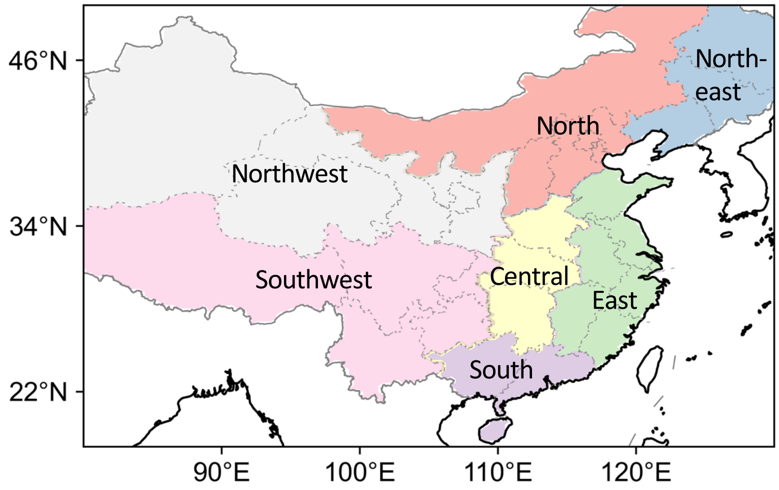

Fig. 3 Provinces contained in the seven subregions of China. North: Beijing, Tianjin, Hebei, Shanxi, Inner Mongolia; Northeast: Heilongjiang, Jilin, Liaoning; East: Shanghai, Jiangsu, Zhejiang, Anhui, Jiangxi, Shandong, Fujian; South: Guangdong, Guangxi, Hainan; Central: Henan, Hubei, Hunan; Southwest: Chongqing, Sichuan, Guizhou, Yunnan, Xizang; Northwest: Shaanxi, Gansu, Qinghai, Ningxia, Xinjiang.

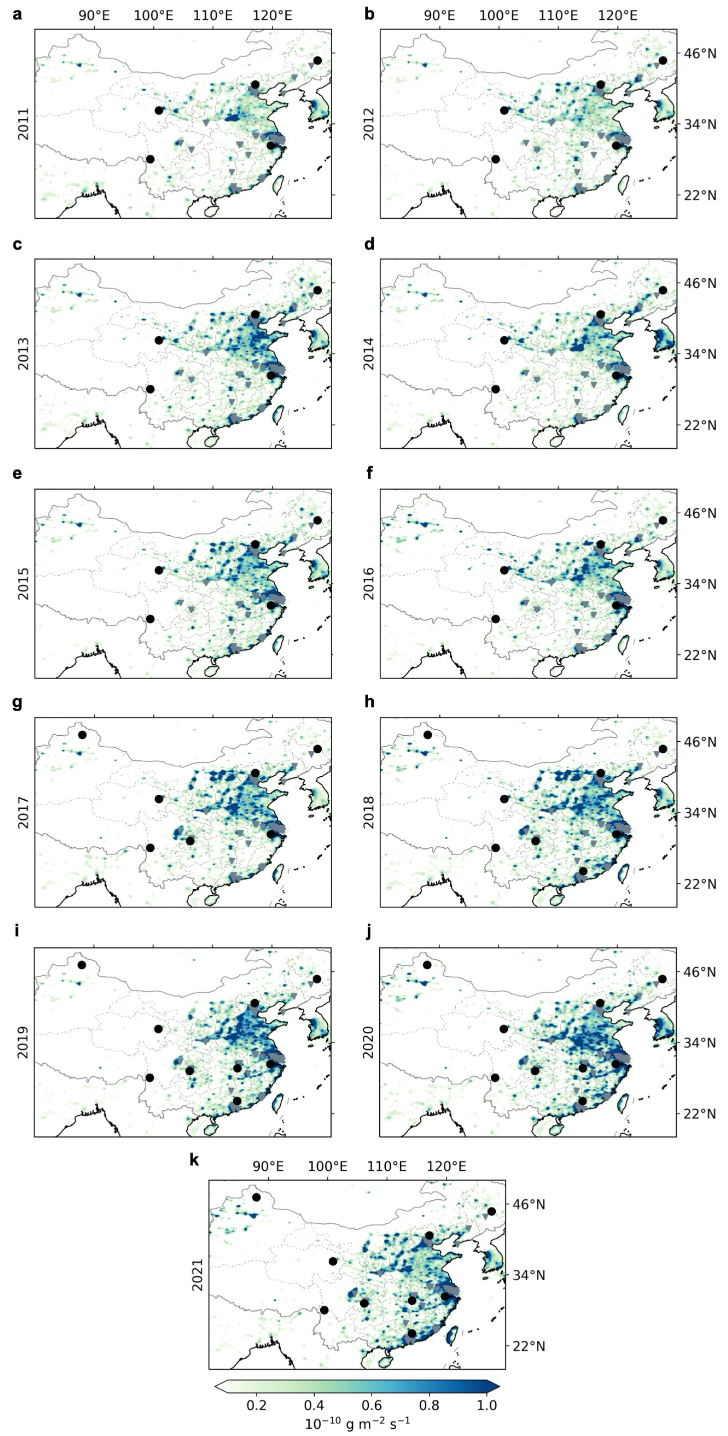

Fig. 4 Spatial distributions of SF_6 emissions in China. The plot for each year is shown in **(a-k)**. Black dots indicate the locations of the active measurement sites in the year. The grey triangles on the plots are the semiconductor factories in China, the locations of which were obtained from Wikipedia (https://en.wikipedia.org/wiki/List_of_semiconductor_fabrication_plants, last access: 16 April 2023).

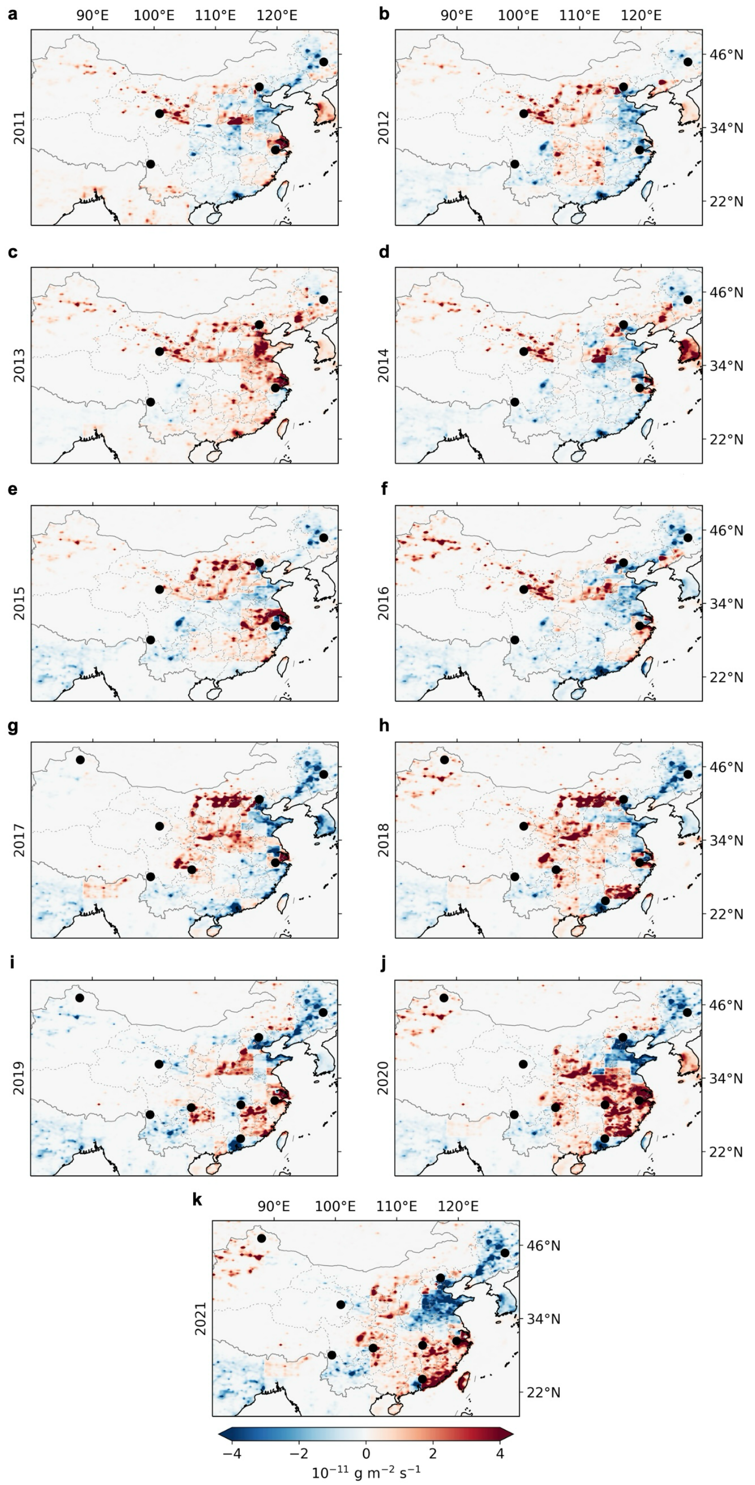

Fig. 5 Difference between a posteriori emissions and a priori nightlights-distributed emissions (a posteriori minus a priori). The plot for each year is shown in **(a-k)**. Black dots indicate the locations of the active measurement sites in the year. These plots indicate that, based on the observations, in most of the years, the inversion process has

scaled up the SF_6 emissions in the western regions (especially the northwest regions) beyond the initial estimates provided by the EDGAR-nightlights a priori emissions, which is already a reasonable proxy for SF_6 emissions in China as indicated in the main text.

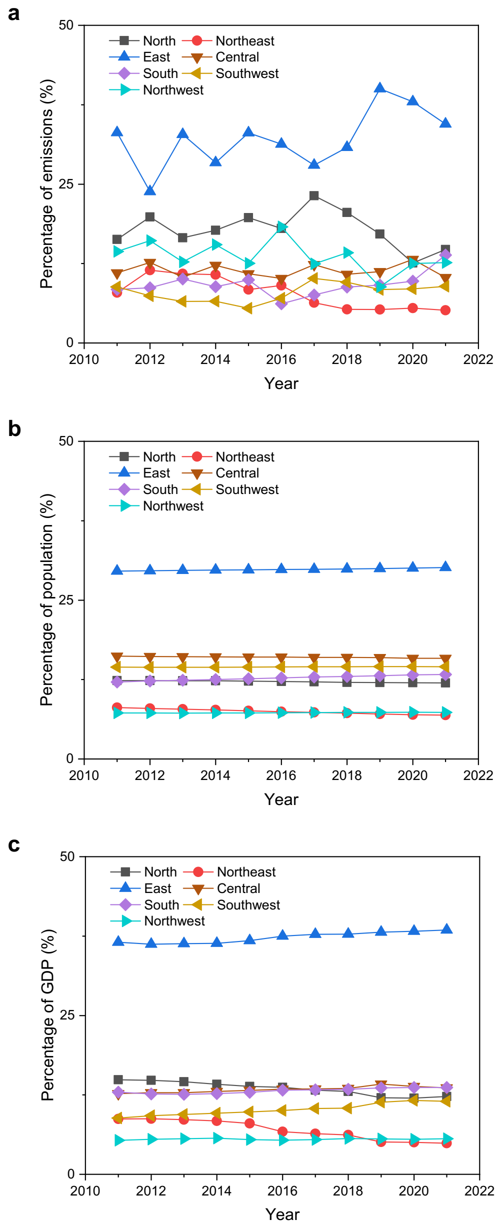

Fig. 6 Contribution of each subregion in China to national total SF_6 emissions, population and gross domestic product (GDP). a Derived SF_6 emissions in each subregion as percentage of the national total emissions in China. **b** Population in each subregion as percentage of the national total population. **c** GDP in each subregion as percentage of the national total GDP. Population and GDP data were obtained from the Yearbook[2]. We can see that the percentage of the population or GDP in each subregion may be different from the percentage of the corresponding SF_6 emissions. For example,

~14% of total SF_6 emissions in China come from the northwest, averaged over 2011-2021, compared to ~7% for its population and ~6% for its GDP.

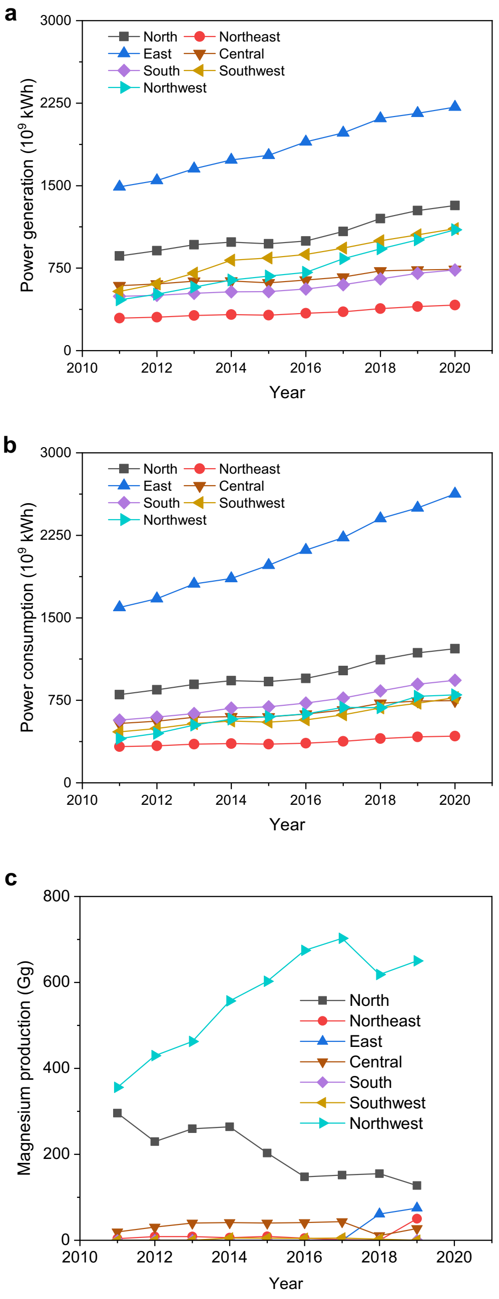

Fig. 7 Annual power generation (a), power consumption (b) and magnesium production (c) in each subregion. The power generation and consumption were obtained from the China Electric Power Yearbook[3] and the China Statistical Yearbook[2]. The magnesium production was obtained from the Yearbook of the Nonferrous Metals Industry of China[4].

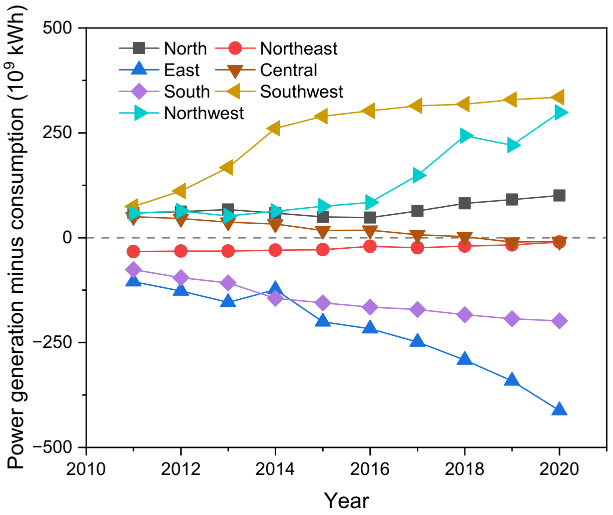

Fig. 8 Electricity imbalance in each subregion. The electricity imbalance was calculated by the power generation[3] minus the power consumption[2]. This imbalance causes a demand in electricity transmission from the northern and western regions to the southern and eastern regions.

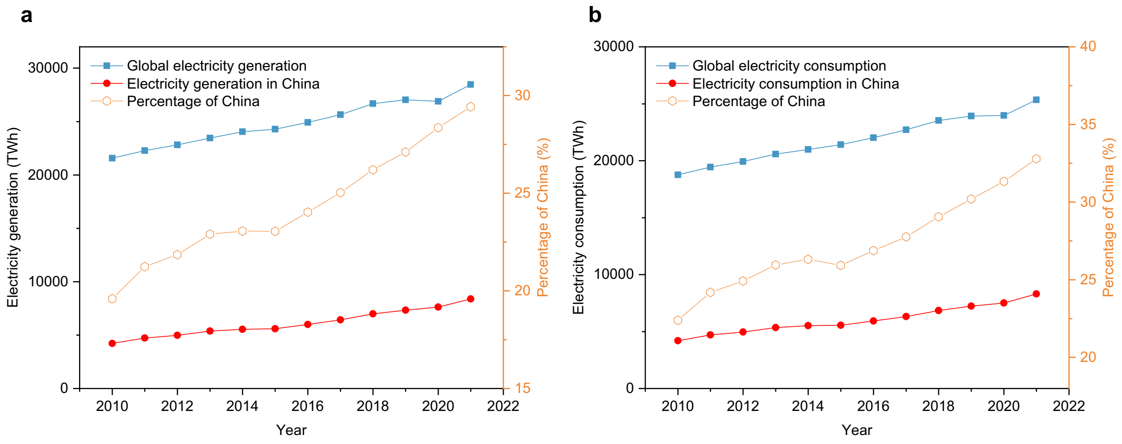

Fig. 9 Electricity generation (a) and consumption (b) in China and globally. The values shown in this plot are obtained from https://www.statista.com (last access: 16 July 2023), where the values for China exhibit a very slight difference compared to the sum of values in each subregion obtained from the Yearbook[2,3], which are shown in Supplementary Fig. 7.

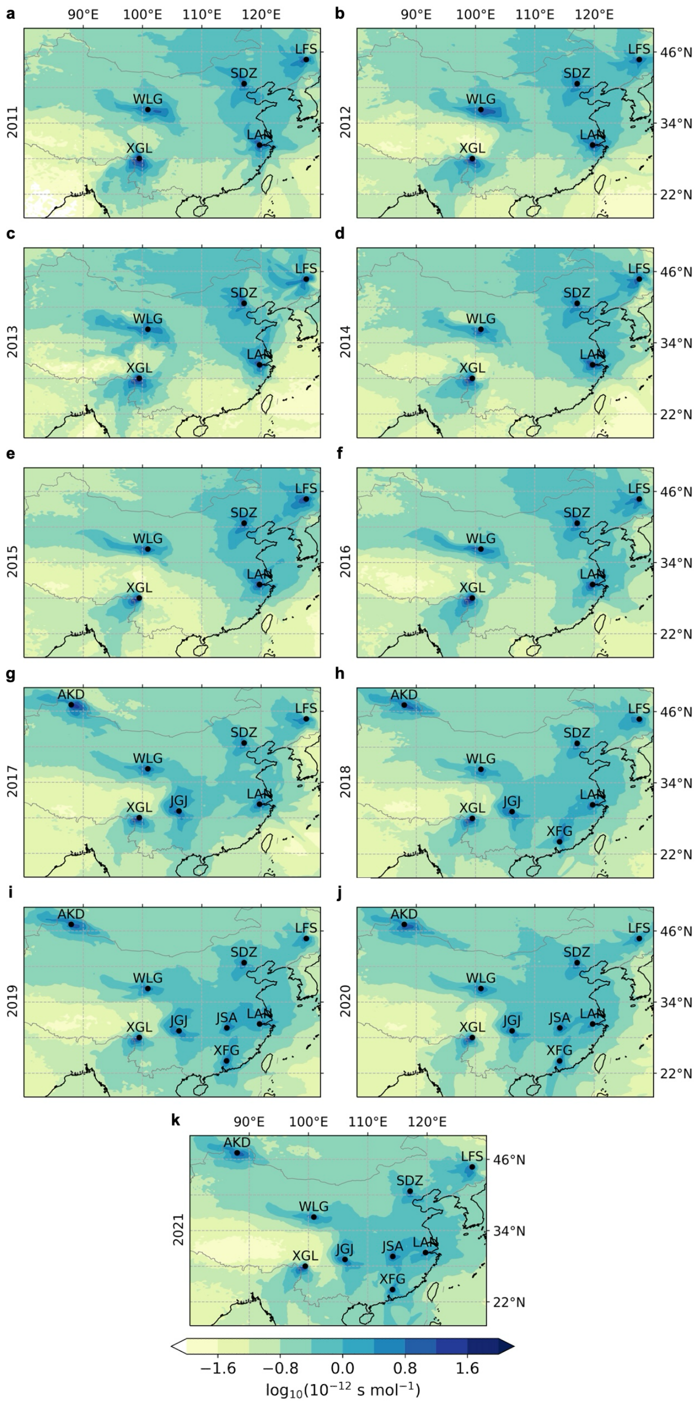

Fig. 10 Sensitivities (footprints) of measurements from the nine sites to emissions fluxes in China. The mean footprint from each site derived by the NAME model was averaged for each year of 2011-2021 and is shown in **(a-k)**. The footprints have good coverage to emissions in China and do not exhibit obvious variation over the period. The measurements have a lower sensitivity to some regions in the southwest of China (mainly

Xizang province), where we do not anticipate large anthropogenic SF_6 emissions. The uncertainty reductions in the southwest of China are reasonably high (as shown in Supplementary Data 2), suggesting that the derived emissions in this region are well constrained.

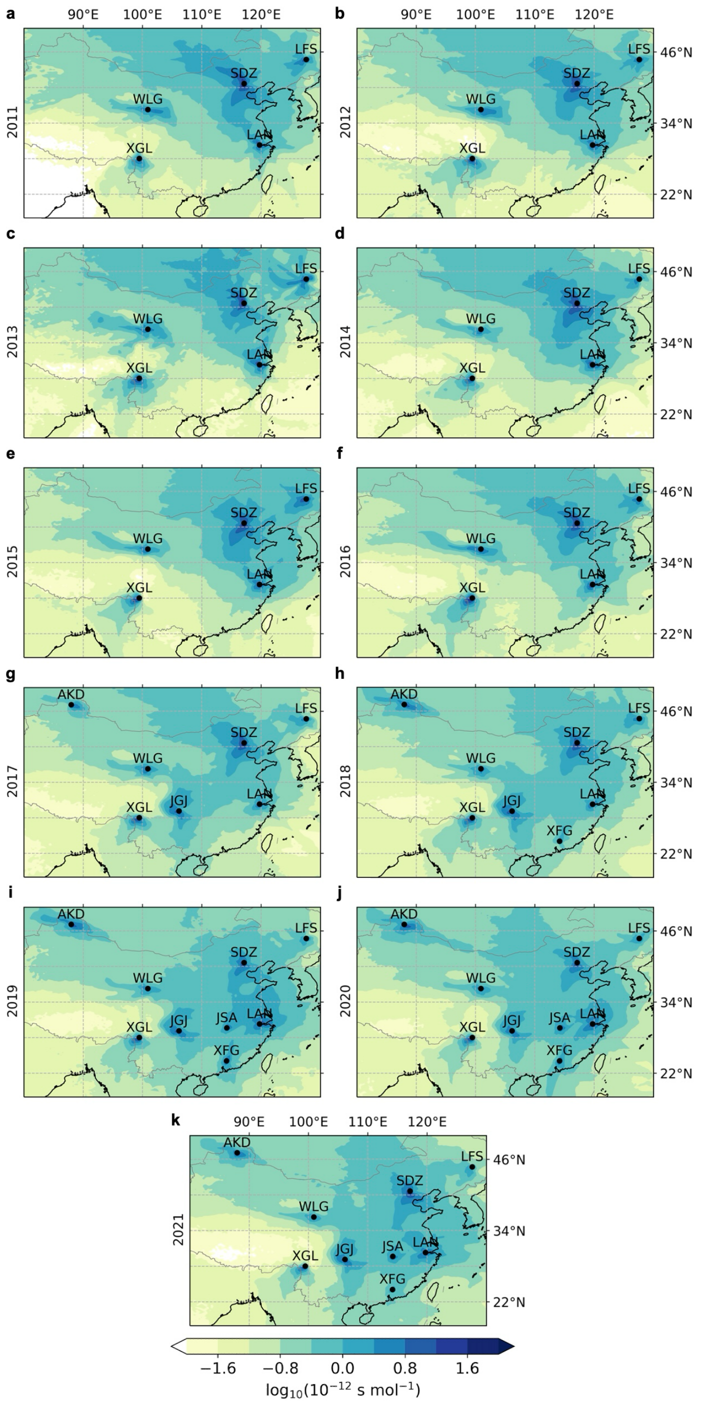

Fig. 11 Sensitivities (footprints) of measurements to emissions fluxes in China. As Supplementary Fig. 10 but the footprints for each year of 2011-2021 shown in **(a-k)** in this plot were weighted by the number of measurements from each site. These sensitivities show similar patterns to Supplementary Fig. 10.

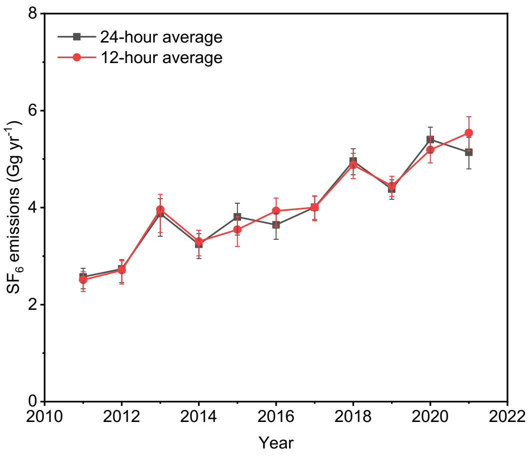

Fig. 12 Emissions derived using 12-hour or 24-hour averaging interval for SDZ in situ data. Either averaging by "12-hour" or "24-hour" does not result in significant differences to the a posteriori results in this study. The error bars represent the 68% uncertainty intervals.

Supplementary Table 1 Improvements in RMSEs between modelled and observed mole fractions for all sites after the inversion

	AKD	JGJ	JSA	LAN	LFS	SDZ	SDZECD	SDZOL	WLG	XFG	XGL	all
2011				14.35%	43.68%	6.86%		11.17%	4.50%		56.43%	12.37%
2012				23.53%	13.27%	17.96%		7.83%	8.60%		49.69%	12.61%
2013				2.96%	12.55%	-8.10%	6.14%		20.13%		20.66%	4.59%
2014				8.09%	20.09%	0.97%	4.35%		-0.91%		62.50%	4.14%
2015				23.76%	33.18%	0.59%	5.14%		-8.29%		60.38%	7.32%
2016				3.25%	49.15%	1.06%		1.18%	-0.82%		46.17%	2.15%
2017	17.17%	5.78%		7.25%	61.90%	-0.29%	2.02%		-0.64%		42.73%	4.22%
2018	15.40%	5.43%		14.57%	57.12%	2.23%		4.60%	-3.38%	22.68%	21.52%	6.73%
2019	26.10%	3.76%	3.08%	4.33%	34.87%	17.15%		7.63%	2.52%	20.35%	64.55%	6.16%
2020	5.29%	4.65%	10.95%	0.86%	22.53%	23.30%		25.36%	-1.32%	19.64%	8.34%	3.23%
2021	4.96%	3.76%	0.59%	6.30%	35.49%	-0.69%		19.84%	-0.60%	15.77%	33.28%	6.65%
all	8.79%	4.74%	3.79%	3.96%	36.28%	4.89%	3.59%	10.55%	0.93%	19.45%	36.52%	5.21%

Supplementary Table 2 Improvements in correlations between modelled and observed mole fractions for all sites after the inversion

	AKD	JGJ	JSA	LAN	LFS	SDZ	SDZECD	SDZOL	WLG	XFG	XGL	all
2011				23.51%	40.59%	6.60%		4.78%	-10.27%		18.00%	9.92%
2012				10.35%	7.45%	28.20%		9.28%	11.18%		8.72%	14.02%
2013				12.32%	52.15%[b]	-4.68%[c]	1.19%		5.95%		28.21%[b]	1.50%
2014				15.42%	9.76%	13.37%	6.67%		3.71%		2.00%	7.04%
2015				24.77%	16.24%	-0.17%	11.64%		-11.30%		18.57%	9.22%
2016				7.02%	25.17%	6.12%		4.08%	0.84%		8.75%	4.14%
2017	-5.50%[a]	6.24%		27.65%	47.70%	0.73%	7.16%		1.61%		15.68%	9.52%
2018	8.55%	10.09%		36.12%	37.41%	0.81%		4.81%	0.28%	14.37%	3.16%	9.79%
2019	2.89%	3.73%	7.76%	8.23%	8.13%	3.36%		3.23%	36.21%[a]	278.43%[b]	15.08%	8.08%
2020	24.74%	1.78%	4.33%	-3.95%	11.50%	10.86%		-0.41%	-0.76%	32.69%	3.62%	15.89%
2021	-11.78%	1487.50%[a]	-5.83%[a]	42.18%	507.62%[a]	4.62%		3.61%	-2.17%	24.05%[b]	19.77%	27.62%
all	1.51%	4.31%	5.61%	6.54%	2.32%	2.13%	7.19%	2.43%	0.37%	56.01%	0.56%	4.71%

a) The correlations obtained using both a posteriori emissions and a priori emissions are not significant (p>0.05).

b) The correlations obtained using a priori emissions are not significant (p>0.05), but those obtained using a posteriori emissions are significant (p<0.05).

c) The correlation obtained using a priori emissions is significant (p<0.05), but that obtained using a posteriori emissions is not significant (p=0.055).

Supplementary Table 3 Site location and sampling frequency

Site name	Short name	Latitude (° N)	Longitude (° E)	Altitude (masl)[a]	Sampling height (magl)[b]	Sampling period	Sampling frequency
Akedala	AKD	47.10	87.97	562	50	2017-2021	Weekly
Mt. Waliguan	WLG	36.29	100.90	3816	80	2011-2021	Weekly
Longfengshan	LFS	44.73	127.60	330	80	2011-2021	Weekly
Shangdianzi	SDZ	40.65	117.12	293	10/80[c]	2011-2012, 2016-2021	In situ GC/MS
						2011-2020	In situ GC-ECD
						2011-2021	Weekly
Jinsha	JSA	29.64	114.21	750	50	2019-2021	Weekly
Lin’an	LAN	30.30	119.73	138	50	2011-2018	Weekly
						2019-2021	Daily
Jiangjin	JGJ	29.15	106.15	262	10	2017-2021	Daily
Shangri-La	XGL	28.01	99.44	3580	50	2011-2021	Weekly
Xinfeng	XFG	24.08	114.17	870	50	2018-2021	Weekly

a) The “masl” represents meters above sea level.

b) The “magl” represents meters above ground level.

c) Shangdianzi changed its sampling height from 10 m to 80 m on August 19th, 2011.

Discussion 1 Uncertainties in posterior emissions using different prior emissions

In the Bayesian inference framework, the posterior values are informed by both observations and prior information. Choices in the prior emissions in the inversion constitute another form of uncertainty in the posterior emissions in addition to the error terms included in the inverse modelling (model-measurement uncertainty). These uncertainties resulting from different prior emissions are not incorporated in the uncertainty intervals expressed in the main text (i.e. the defined 68% uncertainty intervals), but are discussed separately here and in the main text.

In the hierarchical Bayesian inference framework in this study, a log-normal distribution with shape parameters μ=0.2 and σ=0.8 was used as the prior probability distributions for the emissions, which is a non-Gaussian and non-symmetric distribution, and constrains the posterior emissions to positive values. This probability distribution also constrains the posterior emissions to a fairly precise magnitude, due to its relatively narrow probability spread. Thus, the posterior emissions will be largely informed by the prior magnitudes. Inversions using different a priori emission magnitudes, and different prior probability distributions were done to test the robustness of the posterior emission results, as detailed in Supplementary Fig. 13. It can be seen that, the derived emissions using different a priori magnitudes are relatively consistent, both in terms of their a posteriori magnitudes and general trends, even using a very informative prior probability distribution (the log-normal). The derived emissions from different prior probability distributions (truncated-normal with various uncertainty intervals, and log-normal) are also consistent.

Slightly larger discrepancies in the a posteriori emissions derived using different prior magnitudes and probability distributions were observed for 2013 (~1.06 Gg yr^{-1} between the maximum and minimum emissions) and 2021 (up to ~1.15 Gg yr^{-1}) (Supplementary Fig. 13). It is worth noting that there were limited number of observations in 2013 (Supplementary Fig. 1, 2013 has the fewest observations of any year), meaning that the posterior emissions in 2013 are more influenced by the prior emissions (and less impacted by the observations) than in other years. It is also worth noting that there were larger differences between the prior mean emissions of all the runs for 2021 (Supplementary Fig. 13) than other years. These could have contributed to the relatively

large discrepancies in the a posteriori emissions between all the runs in 2013 and 2021. However, the differences between the prior mean emissions of all the runs in the two years have been substantially reduced after the inversion when examining the a posteriori emissions, providing evidence for the effectiveness of the inversion in constraining emissions. For example, the difference in the prior mean emissions in 2021 between the run of “prior: EDGAR; truncated-normal: mu=1, sd=2” and the run of “prior: 3 Gg yr^{-1}; log-normal: mu=0.2, sigma=0.8” in Supplementary Fig. 13 is ~5.2 Gg yr^{-1} (accounting for both a priori emission magnitude and prior probability distribution), while the difference in the corresponding a posteriori emissions between the two runs is ~1.15 Gg yr^{-1}, only ~22% of the prior difference.

Although the a posteriori emission magnitudes and their general trends are relatively robust, the different prior emission information do influence the posterior emission increase between 2011-2013 and 2019-2021. The changes in the posterior emission increase between the two periods when different prior emission information was used would be significant if they are compared to the global total increase (see main text Fig. 3b, global total increase of 1.04 Gg yr^{-1}). However, as discussed in the Main text Fig. 3b, the posterior emission increase between the two periods in China would still be larger than or comparable to the global total increase even if the minimum posterior emission increase among all the runs in Supplementary Fig. 13 (1.15 Gg yr^{-1} from the run of “prior: 4 Gg yr^{-1}; log-normal: mu=0.2, sigma=0.8”) was used.

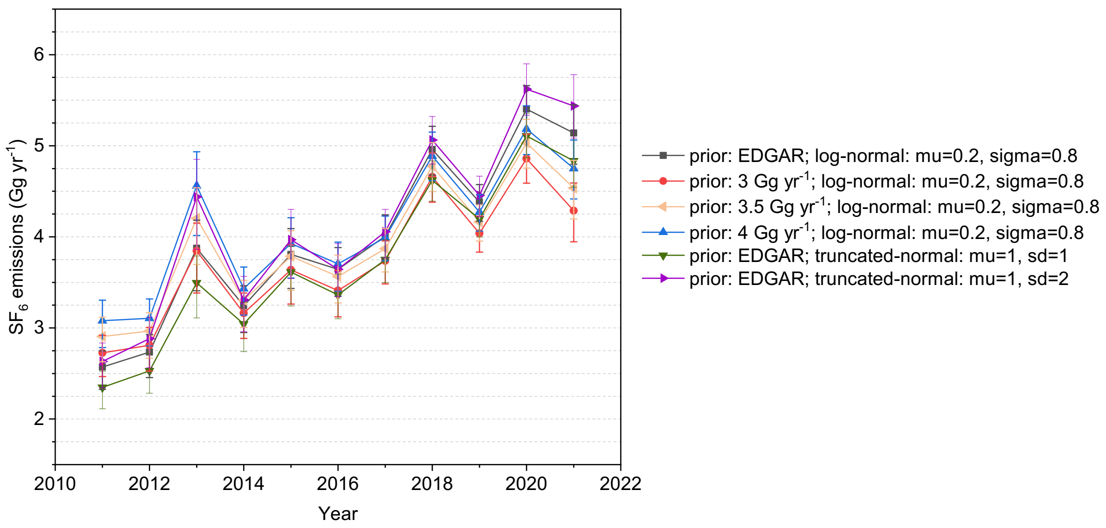

Fig. 13 Estimated emissions in China using different prior emissions. The emissions derived using different a priori emission magnitudes including the EDGAR v7.0 inventory[5] (black, dark green and purple line), and a constant a priori emissions throughout the period (red line with 3 Gg yr^{-1}, orange line with 3.5 Gg yr^{-1} and blue line with 4 Gg yr^{-1}) are shown in the plot. Also shown in the plot are emissions derived using different prior emission probability distributions including the log-normal distribution as used in the main text (black, red, orange and blue line,), as well as truncated-normal distributions with different uncertainties (bounded at 0, dark green line for 100% uncertainty and purple line for 200% uncertainty per basis function). The error bars represent the 68% uncertainty intervals of the derived emissions.

Discussion 2 Quantification of SF_6 emissions in the western regions

In this study, a Chinese network consisting of nine sites including two sites in the northwest (AKD and WLG) and two sites in the southwest (JGJ and XGL) was used to quantify SF_6 emissions in China. These sites provide good sensitivities to most regions of China including the western regions (defined as the northwest China and southwest China, the same hereinafter in this section), as indicated in Supplementary Fig. 10-11, which allows us to quantify SF_6 emissions in China and each subregion. The inversions provide substantial uncertainty reductions, as defined in the Methods section, for SF_6 emissions in China (45-77% reduction for the 68% uncertainty), and in the western regions (18-75% reduction for the 68% uncertainty) (see uncertainty reductions in Supplementary Data 2).

As noted in the main text, the a priori emissions we used in the inversion (EDGAR inventory distributed by nightlights) are good proxies for SF_6 emissions in China. The modelled measurements, using the a posteriori emissions after the inversion, provide a better fit to the real observations beyond these a priori emissions in terms of RMSE or correlation (see Supplementary Table 1-2). In some of the years, there was no improvement in correlation or RMSE after the inversion for WLG, which could be due to the mole fractions from this site generally representing background conditions and that the a priori emissions generally represent any local emissions well. During the inversion, there was a change between the a priori and a posteriori emissions for the western regions (particularly the northwest) (see Supplementary Fig. 5), indicating that the derived emissions in the western regions were informed by the observations.

We conducted a series of test inversions, in which no measurements from sites in western regions of China were used (i.e. excluding measurements from AKD and WLG in the northwest and JGJ and XGL in the southwest) (Supplementary Fig. 14). The differences in the derived a posteriori emissions between runs using different priors (4 Gg yr^{-1} prior and 3 Gg yr^{-1} prior) were used as a measure of how well the observation information used in the inversion constrain the posterior emissions. As indicated in Supplementary Fig. 14, the differences between a posteriori SF_6 emissions in China using different priors will increase by an average of ~71% during 2011-2021 if no sites in the western regions are included (Supplementary Fig. 14b compared to 14a). For the derived SF_6 emissions in

western China and northwest of China, the changes in a posteriori emissions driven by different priors will increase by an average of ~133% (Supplementary Fig. 14d compared to 14c for western) and ~111% (Supplementary Fig. 14f compared to 14e for northwest), respectively. The a posteriori emissions derived with different priors when including all the sites (the left panels of the Supplementary Fig. 14), exhibit greater consistency with each other, compared to the emissions derived without the western sites (the right panels of the Supplementary Fig. 14), which are more driven by the a priori emission magnitudes. Thus, although the sites such as SDZ not in the western regions of China provide some sensitivity to emissions in the western part of China (Supplementary Fig. 10-11), the incorporation of the four sites in the western regions of China significantly contributes to constraining the posterior emissions to an appropriate magnitude. In addition, the uncertainty reductions of the inversion without using the western sites will mostly become lower compared to those of the inversion using all the sites (Supplementary Data 2), especially in the western regions. Excluding the four western sites in the inversion will lead to very small uncertainty reductions in the western regions in some of the years.

In summary, the measurement network used in this study, including the four sites in the western regions of China, enables a relatively accurate quantification of SF_6 emissions in China and its western regions. However, the uncertainty reductions in the northwest regions are somehow lower than in other regions, and there sometimes may not be substantial improvements in the fit to the observations after the inversion. These could be due to the limited number of available measurement sites, as well as infrequent flask sampling in these regions. Denser and more frequent sampling in the western regions would better constrain SF_6 emissions in these crucial areas.

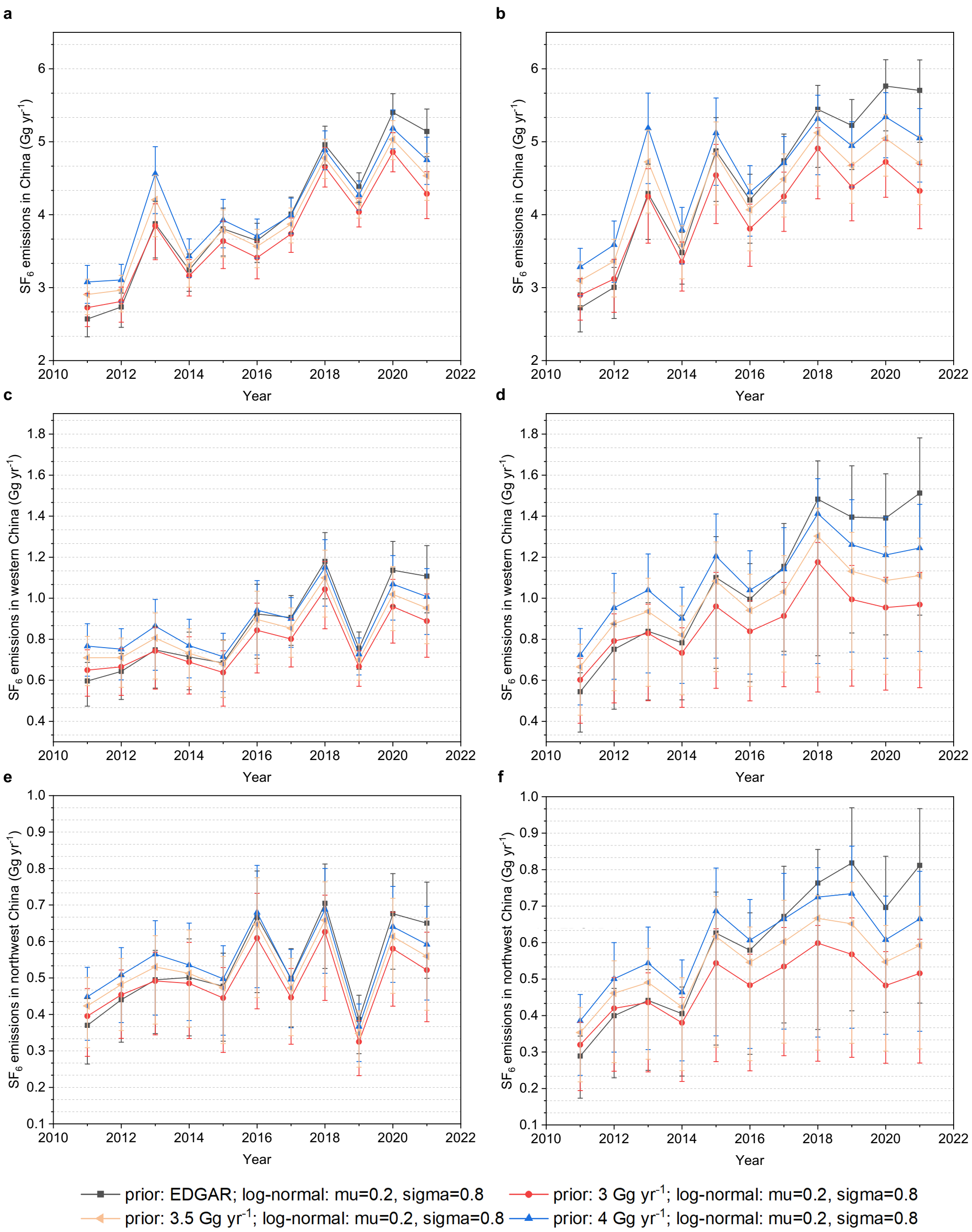

Fig. 14 Derived SF_6 emissions with and without the four sites in the western regions. The four sites are AKD and WLG in the northwest of China, and JGJ and XGL in the southwest of China. The left panels show the results derived using the four sites in western China, and the right panels are the emissions derived without the four sites. Each of the plot presents the derived SF_6 emissions in a specific region using different a priori emissions: **(a-b)** SF_6 emissions in China; **(c-d)** SF_6 emissions in western China

(northwest plus southwest); **(e-f)** SF_6 emissions in the northwest of China. The error bars are the 68% uncertainty intervals of the derived emissions.

Discussion 3 The correlation between SF_6 emissions and the relevant industries and parameters

Pearson correlation coefficients between the derived SF_6 emissions in each province and the power industry[2,3], magnesium industry[4], population[2], GDP[2] and nightlight values[6] in the same province were calculated and the Pearson "*r*" values are shown in Supplementary Table 4. To elucidate the impact of the a priori emission spatial distribution (nightlights-distribution) on the correlation, a sensitivity test was conducted. This test utilized a 'flat prior', where the a priori emissions were uniformly distributed in space in China, and a provincial basis function, where each province served as the fundamental unit in the inverse modelling. The Pearson "*r*" values for the 'flat prior' test run are shown in Supplementary Table 5.

Supplementary Table 4 Correlation between SF_6 emissions in each province and relevant industries and parameters

Year	Power generation[3]	Power consumption[2]	Sum of power generation and consumption[2,3]	Nightlights[6]	Population[2]	GDP[2,a]	Magnesium production[4]	Sample size
2011	0.83**	0.84**	0.85**	0.90**	0.72**	0.74**	0.01	31
2012	0.77**	0.77**	0.79**	0.86**	0.64**	0.68**	0.19	31
2013	0.82**	0.88**	0.87**	0.97**	0.76**	0.83**	0.01	31
2014	0.76**	0.85**	0.83**	0.90**	0.71**	0.73**	0.06	31
2015	0.80**	0.87**	0.86**	0.91**	0.70**	0.76**	0.05	31
2016	0.74**	0.79**	0.79**	0.84**	0.59**	0.59**	0.06	31
2017	0.84**	0.79**	0.84**	0.82**	0.66**	0.62**	0.18	31
2018	0.88**	0.83**	0.88**	0.81**	0.65**	0.64**	0.24	31
2019	0.74**	0.88**	0.84**	0.88**	0.77**	0.80**	0.13	31
2020	0.73**	0.83**	0.81**	0.82**	0.76**	0.79**		31
all	0.79**	0.83**	0.83**	0.86**	0.66**	0.73**	0.11	310[b]

** The '*p*' value for the coefficient is <0.01.

a) "GDP" is the Gross Domestic Product in each province (Gross Regional Product).

b) The sample size for the correlation coefficient with magnesium production over all years is 279 due to the lack of magnesium data in 2020.

Supplementary Table 5 Correlation between SF_6 emissions in each province and relevant industries and parameters for the test run with flat prior

Year	Power generation[3]	Power consumption[2]	Sum of power generation and consumption[2,3]	Nightlights[6]	Population[2]	GDP[2,a]	Magnesium production[4]	Sample size
2011	0.19	0.10	0.14	0.16	0.09	-0.03	0.36*	31
2012	0.12	-0.01	0.05	0.03	-0.12	-0.18	0.46**	31
2013	0.29	0.28	0.29	0.39*	0.23	0.26	-0.06	31
2014	0.31	0.21	0.26	0.25	-0.04	-0.02	0.33	31
2015	0.36*	0.26	0.32	0.31	0.10	0.08	0.26	31
2016	0.24	0.14	0.19	0.16	-0.04	-0.11	0.18	31
2017	0.31	0.25	0.29	0.34	0.26	0.11	0.24	31
2018	0.36*	0.26	0.32	0.30	0.15	0.08	0.31	31
2019	0.46*	0.51**	0.50**	0.53**	0.32	0.32	0.07	31
2020	0.43*	0.46**	0.46**	0.48**	0.52**	0.36*		31
all	0.33**	0.28**	0.31**	0.33**	0.16**	0.14*	0.22**	310[b]

* The 'p' value for the coefficient is <0.05.

** The 'p' value for the coefficient is <0.01.

a) "GDP" is the Gross Domestic Product in each province (Gross Regional Product).

b) The sample size for the correlation coefficient with magnesium production over all years is 279 due to the lack of magnesium data in 2020.

Discussion 4 Reduction of CO_2 emissions from renewable energy power generation

The electricity generation using photovoltaic and wind power (kWh) in China during 2011-2020 were obtained from the China Electric Power Yearbook[3] and are shown in Supplementary Table 6. We assume that the electricity generation by photovoltaic and wind power replaces the coal fire power generation. The annual mean increase of electricity generation using photovoltaic and wind power over 2011-2020 was calculated to be 7.46×10^{10} kWh yr^{-1}, by a linear regression. Thus, the annual mean reduction of CO_2 emissions from applying renewable energy to replace coal fire power in electricity generation was estimated to be ~59 Mt yr^{-1}, assuming a CO_2 emission factor of 795.2 g CO_2 per kWh electricity generated by coal fire power plant[7].

Supplementary Table 6 Electricity generation from photovoltaic and wind power in China

Year	Photovoltaic (10^8 kWh)	Wind (10^8 kWh)	Sum (10^8 kWh)
2011	6	741	747
2012	36	1030	1066
2013	84	1383	1467
2014	235	1598	1833
2015	395	1856	2251
2016	665	2409	3074
2017	1166	3034	4200
2018	1769	3658	5427
2019	2240	4053	6293
2020	2611	4665	7276

Supplementary References

1. Simmonds, P. G. et al. The increasing atmospheric burden of the greenhouse gas sulfur hexafluoride (SF_6). *Atmos. Chem. Phys.* **20**, 7271–7290 (2020).
2. National Bureau of Statistics of China. *China Statistical Yearbook*. (China Statistics Press, Beijing, 2021).
3. Editorial board of China Electric Power Yearbook. *China Electric Power Yearbook* (in Chinese). (China Electric Power Press, Beijing, 2021).
4. China Nonferrous Metals Industry Association. *The Yearbook of Nonferrous Metals Industry of China* (in Chinese). (China Nonferrous Metals Industry Yearbook Press, Beijing, 2020).
5. EDGAR (Emissions Database for Global Atmospheric Research). Community GHG Database version 7.0, European Commission, JRC (Datasets). https://edgar.jrc.ec.europa.eu/dataset_ghg70 (2022).
6. NOAA's National Geophysical Data Center. Version 4 DMSP-OLS Nighttime Lights Time Series. https://www.ngdc.noaa.gov/eog/data/web_data/v4composites/ (last access: 1 March 2021).
7. Tong, D. et al. Current emissions and future mitigation pathways of coal-fired power plants in China from 2010 to 2030. *Environ. Sci. Technol.* **52**, 12905–12914 (2018).
