# Supplementary material for: Sustained growth of sulfur hexafluoride emissions in China inferred from atmospheric observations
Source: Nat Commun. 2024 Mar 5;15:1997. doi: 10.1038/s41467-024-46084-3 (PMC10915133; doi:10.1038/s41467-024-46084-3)
Supplement: Supplementary file 3 — Description of Additional Supplementary Files [file 41467_2024_46084_MOESM3_ESM.pdf]

Description of Additional Supplementary Files:

Supplementary Data 1: Observations of SF_6 mole fractions at nine Chinese sites that were used in this study to derive SF_6 emissions in China.

Supplementary Data 2: Uncertainty reductions from the regional inversion.

Supplementary Data 3: Derived SF_6 emissions in China. Emissions in each province/ subregion and total emissions in China are provided.
